# Atypical Pityriasis rosea in a black child: a case report

**DOI:** 10.1186/1757-1626-2-6796

**Published:** 2009-04-29

**Authors:** Sergio Vano-Galvan, Dong-Lai Ma, Alejandro Lopez-Neyra, Bibiana Perez, Ernesto Muñoz-Zato, Pedro Jaén

**Affiliations:** 1 Department of Dermatology, Ramon Y Cajal Hospital, University of Alcala, Madrid, Spain, Carretera Colmenar km 9.100 28034 Madrid, Spain; 2 Department of Dermatology, Peking Union Medical College Hospital, Chinese Academy of Medical Sciences and Peking Union Medical College, Beijing 100730, PR China; 3 Department of Paediatrics, Niño Jesus Hospital, Avda, Menéndez Pelayo 65, 28009, Madrid, Spain

## Abstract

**Introduction:**

Pityriasis rosea is a self-limited inflammatory condition of the skin that mostly affects healthy children and adolescents. Atypical cases of Pityriasis rosea are fairly common and less readily recognized than typical eruptions, and may pose a diagnostic challenge.

**Case presentation:**

We report the case of a 12-year-old black child that developed an intense pruritic papular eruption with intense facial involvement that was diagnosed of Pityriasis rosea and resolved after five weeks leaving a slight hyperpigmentation.

**Conclusion:**

Facial and scalp involvement, post-inflammatory disorders of pigmentation and papular lesions are characteristics typically associated to black patients with Pityriasis rosea. The knowledge of features found more frequently in dark-skinned population may be helpful to physicians for diagnosing an atypical Pityriasis rosea in these patients.

## Introduction

Pityriasis rosea (PR) is a self-limited inflammatory condition of the skin that mostly affects healthy children and adolescents. Typically, a maculopapular eruption with an elevated scaly border located on trunk and proximal extremities is observed [1,2]. PR in black children appears to be more florid and has a greater tendency to overstep the classic boundaries of distribution on the body, to affect face and scalp [3].

We report a case of a black child with a generalized papular pruritic eruption with intense facial involvement that we diagnosed as an atypical PR. We discuss the association between some features of atypical PR and dark-skinned population.

## Case Presentation

A 12-year-old black healthy child, born in Colombia, was referred to our department with a 2-week history of generalized and intense pruritic eruption predominantly involving face, neck and back. His mother had noticed one lesion on his left shoulder five days before the generalized eruption. Neither fever nor preceding coryzal symptoms were found. Patient denied any history of prior eruptions or use of medication.

**Figure 1:**
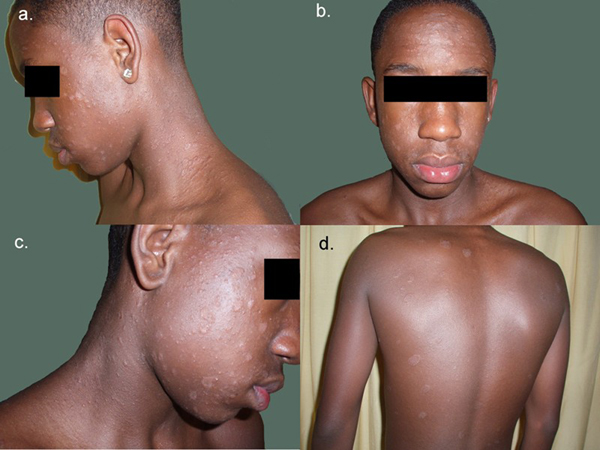
**Lesions involving the face and orientated along lines of skin cleavage**.

Dermatological examination revealed multiple small oval papules 0.5-1 cm in diameter distributed on face and neck and some circular slightly scaly patches 4-5 cm in diameter over his back (Figure 1). Some lesions were orientated along lines of skin cleavage. Mucosal surfaces, palms, soles and scalp were normal.

Because of the atypical morphology and distribution of the rash, a skin biopsy and a stain for fungi were taken. Laboratory analyses and stain for fungus were normal. Serologic tests for syphilis (RPR, FTA-Abs) were negative. Histological examination showed mild spongiosis and focal parakeratosis. A superficial perivascular infiltrate composed of lymphocytes and histiocytes was observed in the dermis.

Suspecting an atypical PR, oral dexclorfeniramine and topical mentol lotion were prescribed in order to attenuate pruritus.

Five weeks later, lesions had resolved spontaneously leaving a slight hyperpigmentation within the affected areas that persisted after 6 months of follow-up.

Based on the morphology of lesions, the course of eruption and the compatible histological findings, we believe the most likely diagnosis in our patient was atypical PR.

## Discussion

PR is a self-limiting, inflammatory skin disease of acute or subacute evolution. PR may present with a wide spectrum of variants that make the diagnosis difficult for the physician. We would like to emphasize the relationship between some features of atypical PR and dark-skinned children. Our patient developed an intensely pruritic eruption with papular lesions and important facial involvement. Face is not usually affected in PR [2,4]. However, in black patients PR involves more frequently this area [3,5,6]. In addition, literature also states that black children with PR seem to have more frequent papular lesions and more sequelae of pigmentation, like our patient [2,3,5]. Amer et al. [3] studied 50 black American children with PR; they found 30% of facial involvement, more frequent papular lesions and pigmentary changes in 62 %. The presence of pruritus was also observed in nearly all of their patients.

In summary, atypical cases of PR are fairly common and less readily recognized than typical eruptions, and may pose a diagnostic challenge. Facial and scalp involvement, post-inflammatory disorders of pigmentation and papular lesions are characteristics typically associated to black patients with PR [3]-[7]. In addition, presence of intense pruritus may be observed in black children and it must not rule out the diagnosis of PR [3]. Knowledge of features found more frequently in dark-skinned population may be helpful to physicians for diagnosing an atypical PR in these patients.

## List of abbreviations

PR: pityriasis rosea; RPR: "Rapid Plasma Reagin" (syphilis screening test); FTA-Abs: "Fluorescent Treponemal Antibody Absorbed" (syphilis screening test).

## Consent

"Written informed consent was obtained from the patient for publication of this case report and accompanying images. A copy of the written consent is available for review by the Editor-in-Chief of this journal."

## Competing interests

The authors declare that they have no competing interests.

## Authors' Contributions

SVG and BP wrote the initial draft of and helped revise the manuscript. Dr LN obtained consent from the patients and helped revise the manuscript. DLM, EMZ and PJ assisted with manuscript revision. All authors read and approved the final manuscript.
